# Reliability of end-tidal carbon dioxide as a surrogate for arterial carbon dioxide assessment in infants undergoing thoracoscopic surgery with one-lung ventilation

**DOI:** 10.1186/s12871-026-04077-3

**Published:** 2026-07-03

**Authors:** Change Zhu, Sisi Wei, Rufang Zhang, Mazhong Zhang, Rong Wei

**Affiliations:** 1 Department of Anesthesiology, Shanghai Children’s Hospital, Shanghai Jiao Tong University School of Medicine, No. 355 Luding Road, Putuo District, Shanghai, 200062 China; 2 Cardiothoracic Surgery Department, Shanghai Children’s Hospital, Shanghai Jiao Tong University School of Medicine, Shanghai, China; 3 Department of Anesthesiology, Shanghai Children’s Medical Center, Shanghai Jiao Tong University School of Medicine, No. 1678 Dongfang Road, Pudong New Area, Shanghai, 200127 China; 4 Department of Anesthesiology, Bozhou People’s Hospital, Bozhou, Anhui China

**Keywords:** Infant, One–lung ventilation, Thoracoscopy, End–tidal carbon dioxide, Bland–Altman analysis, Lung compliance

## Abstract

**Background:**

The reliability of end-tidal carbon dioxide (EtCO₂) as a surrogate for arterial arterial carbon dioxide partial pressure (PaCO₂) during one-lung ventilation (OLV) in infants is uncertain. This study evaluated EtCO₂-PaCO₂ agreement in infants undergoing OLV and identified factors associated with clinically significant discrepancy.

**Methods:**

This secondary analysis of a prospective, randomized controlled trial included 172 infants (aged ≤ 2 years) undergoing thoracoscopic surgery. Paired PaCO₂ and EtCO₂ measurements were obtained 20 min after initiating OLV. Clinical agreement was defined a priori as an absolute difference of ≤ 5 mmHg. Statistical evaluations included Bland–Altman analysis and multivariable logistic regression to identify predictors of disagreement, and correlation analyses.

**Results:**

During OLV, the mean EtCO₂–PaCO₂ bias was 2.8 mmHg with wide 95% limits of agreement (-4.7 to 10.3 mmHg), exceeding the clinically acceptable threshold. Oxygenation impairment (PaO₂/FiO₂ < 200 mmHg) significantly reduced the proportion of clinically acceptable measurements (55.9% vs. 81.7%, *P* = 0.0001). In multivariable analysis, EtCO₂–PaCO₂ disagreement occurred exclusively in infants ventilated under the non–protective protocol (VT 8 mL/kg with ZEEP). After excluding ventilatory variables that were structurally confounded with randomized allocation, lower lung compliance was independently associated with disagreement (adjusted OR 1.66, 95% CI 1.05–2.64, *P* = 0.030).

**Conclusions:**

EtCO₂ provides only limited reliability as a stand–alone substitute for arterial blood gas analysis in infants during OLV, particularly under non–protective ventilation. The lung–protective ventilation strategy as a composite intervention is associated with substantially improved EtCO₂–PaCO₂ agreement during OLV in infants. Among individual patient-level factors, lower lung compliance independently predicts EtCO₂–PaCO₂ disagreement and may serve as a bedside indicator for clinicians to consider supplementary arterial blood gas analysis.

**Supplementary Information:**

The online version contains supplementary material available at https://doi.org/10.1186/s12871-026-04077-3.

## Introduction

To facilitate surgical access during video–assisted thoracoscopic surgery (VATS), an artificial carbon dioxide (CO_2_) pneumothorax is mandatory. When combined with the requirement for one–lung ventilation (OLV), this procedure places pediatric patients at significant risk for developing severe intraoperative hypercapnia [1]. In infants, uncontrolled hypercapnia is of particular concern as it can disrupt cerebral autoregulation [2], potentially precipitating cerebral edema [3], vascular spasms, and the suppression of cortical electrical activity [4]. Consequently, precise monitoring of arterial carbon dioxide partial pressure (PaCO_2_) is mandated to ensure patient safety. Although arterial blood gas (ABG) analysis is considered the definitive method for CO2 measurement, its clinical utility is limited by its invasive nature and the intermittent nature of sampling. Although EtCO₂ is frequently monitored in clinical practice [5–7], anesthesiologists are generally familiar with the expected physiological difference between EtCO₂ and PaCO₂, rather than viewing it as a perfect surrogate. Theoretically, EtCO2 values are derived from the exhalation of CO_2_–rich alveolar gas mixed with gas from alveolar dead space—regions that are ventilated but not perfused and poorly perfused regions. Under stable physiological conditions with normal ventilation-perfusion (V/Q) matching, the gradient between PaCO_2_ and EtCO_2_ is typically narrow, often cited within the range of 0–5 mmHg [8, 9]. However, the reliability of this gradient is challenged during OLV. The procedure inherently induces significant V/Q mismatching, which may unpredictably increase the dead space fraction [10, 11]. While this phenomenon is well–documented in adults, there is a paucity of data regarding the accuracy of EtCO_2_ monitoring specifically in infants aged less than 2 years during OLV. In this population, unique anatomical and physiological characteristics may further exacerbate the discrepancy between arterial and end–tidal values. To address this knowledge gap, the present study aimed to evaluate the validity of EtCO_2_ as a substitute for PaCO_2_ in infants undergoing OLV and to identify specific risk factors associated with a significant EtCO_2_–PaCO_2_ gradient. This secondary analysis was conducted using data from a previously published clinical trial [12].

## Materials and methods study design and ethical considerations

This secondary analysis of a prospective, randomized controlled trial (ChiCTR2200059270) [12] was conducted and reported in accordance with the STROBE guidelines. The study protocol was approved by the Ethics Committee of Shanghai Children’s Hospital (Approval No. 2021R129-F02). Written informed consent was obtained from the parents or legal guardians of all participants prior to their enrollment in the parent trial. A clinically acceptable limit of agreement between PaCO₂ and EtCO₂ was defined a priori as ± 5 mmHg (1 mmHg = 0.133 kPa), consistent with the parent trial protocol [13]. We acknowledge at the outset that this symmetric threshold reflects a clinical pragmatic convention rather than a physiological standard: under normal conditions, the PaCO₂–EtCO₂ gradient is expected to be positive, and positive versus negative gradients should not be regarded as equivalent phenomena. The threshold was retained in this secondary analysis to preserve interpretive consistency with the prespecified endpoint of the original randomized study, with its conceptual and physiological implications addressed in the Discussion.

### Participants and setting

This secondary analysis was derived from 213 patients who successfully completed a prospective randomized controlled trial [12]. After excluding 41 children older than 2 years, a final cohort of 172 infants undergoing thoracoscopic surgery with OLV was included. Patients from the original trial who were ≥ 2 years old, or those with incomplete paired EtCO₂ and PaCO₂ measurements during OLV, were excluded from this analysis. The study period spanned from May 6, 2022, to August 31, 2023, within the Department of Cardiothoracic Surgery. All participants followed the standardized anesthetic and surgical protocols detailed in our previous work [12]. In this study, “sex” is defined based on the biological attributes assigned at birth (male or female) as recorded in the patients’ electronic medical records.

### Anesthesia and ventilation protocol

Upon entry to the operating theater, standard monitoring (electrocardiography, pulse oximetry, and non–invasive blood pressure) was established. Following routine pre–oxygenation, anesthesia was induced intravenously using propofol (2.0–3.0 mg/kg), sufentanil (1.0–2.0 µg/kg), and either cisatracurium (0.1–0.2 mg/kg) or rocuronium (0.6 mg/kg). For anesthesia maintenance, patients received an inhalation of sevoflurane (1.5–2.0 vol%) combined with continuous intravenous infusions of sufentanil (1.0–2.0 µg/kg/h) and cisatracurium (0.1 mg/kg/h). Following tracheal intubation, an invasive arterial line was placed in the radial artery for hemodynamic monitoring and blood sampling. Lung isolation was achieved using an extraluminal bronchial blocker (Hangzhou Tanpa Medical Technology, Hangzhou, China) positioned under fiberoptic bronchoscopic guidance. The quality of lung isolation was assessed by the surgical team and graded as “good” for all included cases [14]. All patients received ventilation using the Datex–Ohmeda Avance CS2 Anesthesia Machine (GE Healthcare, Madison, WI, USA). Mechanical ventilation was delivered via a pressure–controlled volume–guaranteed (PCV–VG) mode. Ventilatory parameters were initially set with an inspiratory–to–expiratory (I: E) ratio of 1:2. The respiratory rate (RR) was fixed at 20 breaths/min during TLV and 25 breaths/min during OLV to target normocapnia. As dictated by the parent trial protocol, patients were managed with either a non–LPV or an LPV strategy. In the non–LPV group, the tidal volume was set at 10 ml kg⁻¹ during TLV and 8 ml kg⁻¹ during OLV with ZEEP (zero PEEP). In the LPV groups, the tidal volume was limited to 8 ml kg⁻¹ during TLV and 6 ml kg⁻¹ during OLV. Additionally, patients in the LPV groups received either a fixed PEEP of 5 cmH₂O throughout the procedure or an individualized PEEP titrated to achieve the lowest driving pressure during OLV. Data Acquisition and Measurements Data collection occurred at two specific time points: T1:5 min after intubation during TLV (supine position). T2:20 min after the initiation of OLV (lateral decubitus position). At these time points, we recorded hemodynamic variables (invasive blood pressure, heart rate) and ventilator settings (e.g., PEEP, VT, RR) and respiratory mechanics parameters (e.g., peak airway pressure, lung compliance). To prevent ex vivo metabolic alterations in blood gas tensions, all arterial samples were analyzed immediately (strictly within 2 min of collection) using a point–of–care blood gas analyzer located within the operating theater. CO_2_ Measurement and Calculation PaCO_2_ was analyzed using an ABL800 blood gas analyzer (Radiometer) from radial artery samples drawn at T1 and T2. Simultaneous EtCO_2_ readings were obtained using a sidestream carbon dioxide analyzer (D–Fend Pro, GE Healthcare), which was calibrated prior to each case. To rigorously minimize apparatus dead space and prevent sample dilution from the circuit’s bias flow, the EtCO₂ sampling line was connected directly to the Y–piece, and heat and moisture exchangers (HMEs) were deliberately avoided during the procedure. To assess the inefficiency of ventilation, we calculated a highly simplified clinical estimate of the estimated dead–space index. Due to the unavailability of volumetric capnography required to measure mean mixed expired CO₂ for the classic Bohr equation, we utilized a widely accepted clinical surrogate based on the Enghoff modification: estimated VD/VT= (PaCO_2_–EtCO_2_) / PaCO_2_ [15]. We acknowledge that substituting end–tidal CO₂ for mixed expired CO₂ represents a significant physiological simplification, and thus, this calculated value serves as a clinical index of the alveolar dead space trend rather than a precise absolute measurement.

### Study definitions

In clinical practice and based on prior validation literature, a clinically acceptable discrepancy between non–invasive capnography and arterial blood gas is typically defined as ≤ 5 mmHg. Therefore, in this study, ‘clinical agreement’ was defined a priori as an absolute EtCO₂–PaCO₂ gradient of ≤ 5 mmHg. Patients were stratified into ‘Agreement’ and ‘Non–agreement’ groups based on this threshold for comparative analysis. Patients were stratified into two subgroups based on a PaO₂/FiO₂ threshold of 200 mmHg. This threshold was selected a priori as it represents a universally recognized metric for moderate–to–severe oxygenation impairment. Furthermore, based on standard iso–shunt physiological models, a PaO₂/FiO₂ ratio < 200 mmHg correlates mathematically with a substantial intrapulmonary shunt fraction (> 15–20%), representing a clinically significant derangement during one–lung ventilation [16, 17].

Hypotension was based on age–adjusted criteria (defined as a systolic blood pressure < 80+[2×age in years] mmHg).

### Statistical analysis

Data normality was assessed using the Shapiro–Wilk test. Continuous variables are presented as mean ± standard deviation (SD) for normally distributed data or median [interquartile range, IQR] for non–normally distributed data. Categorical variables are expressed as frequencies and percentages (n, %). Method comparison was performed using Bland–Altman analysis to quantify bias and 95% limits of agreement [18], complemented by Deming regression [19] to assess constant and proportional bias under the assumption of measurement error in both variables.

The relationship between the Oxygenation Index and the EtCO₂–PaCO₂ gradient was assessed using Spearman’s rank correlation, given the non-normal distribution of the Oxygenation Index in this infant cohort (Shapiro–Wilk *P* < 0.05) and the non–linear pattern observed on scatter–plot inspection. PaCO₂–EtCO₂ gradients were also analyzed as continuous variables according to ventilation strategy. Their distributions were displayed using box-and-jitter plots with individual paired measurements, and between-group differences were assessed using the Mann–Whitney U test. Reference lines at 0 mmHg and + 5 mmHg were added to indicate no gradient and the predefined disagreement threshold, respectively. The proportions of negative gradients and gradients > 5 mmHg were additionally summarized by ventilation arm.

### Independent predictors of clinical agreement

Multivariable logistic regression analysis was performed to identify independent predictors of clinical agreement (defined as an absolute difference ≤ 5 mmHg between PaCO₂ and EtCO₂). The candidate variables incorporated into the model—including age, gender, surgical side, hypotension, and lung compliance.

Software and Significance General statistical analyses were performed using SPSS version 26.0 (IBM Corp, Armonk, NY, USA). Advanced method comparison analyses (Bland–Altman, and Deming regressions) were conducted using MedCalc^®^ Statistical Software version 19.6.4 (MedCalc Software Ltd, Ostend, Belgium). All statistical tests were two–tailed, and a P–value < 0.05 was considered statistically significant.

## Results

### Study population characteristics

**Fig. 1:**
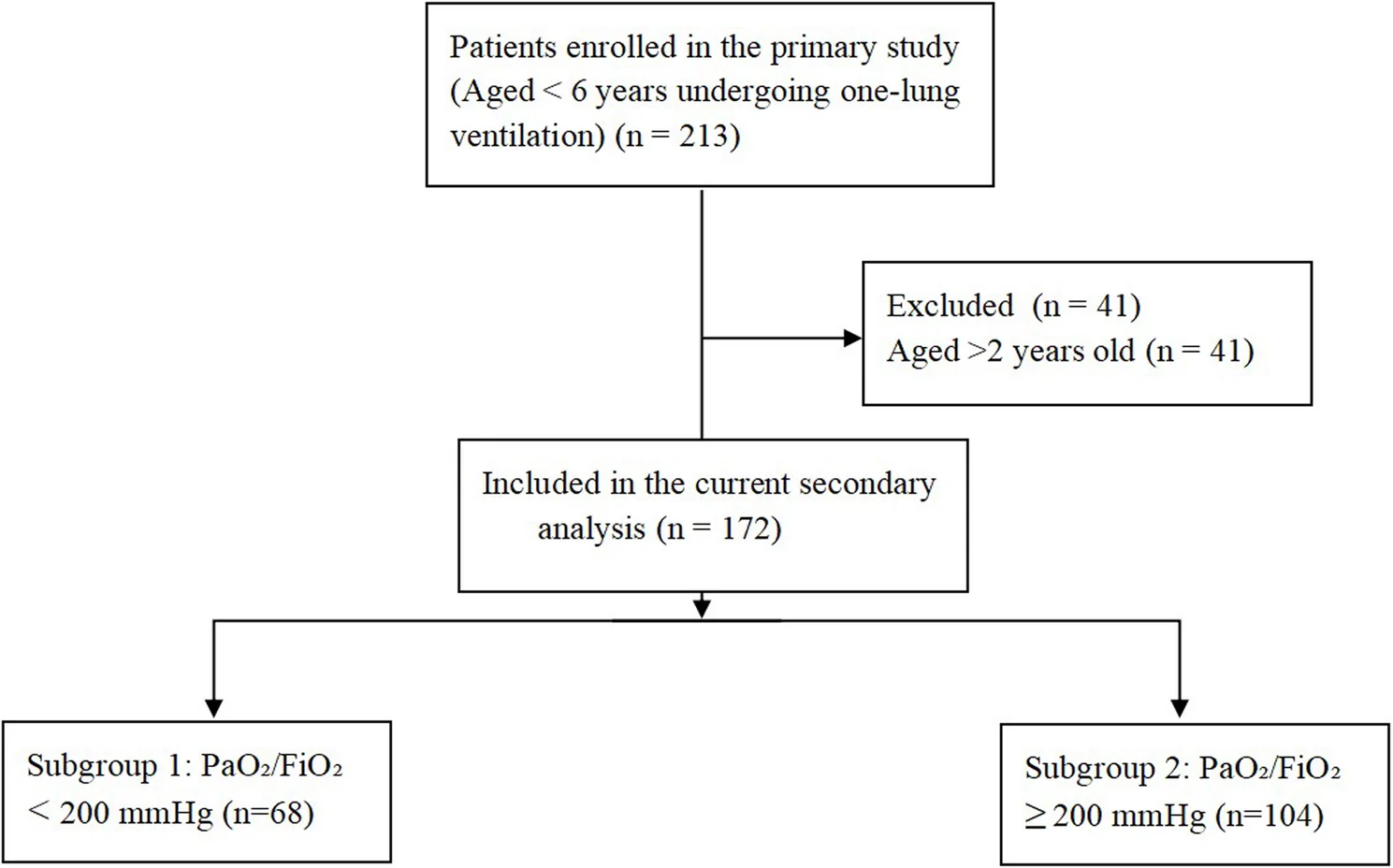
Patient flow diagram

From the original 213 patients enrolled in the parent trial, 41 patients were excluded due to being > 2 years of age, leaving a final cohort of 172 infants for this analysis (Fig. 1).

The cohort consisted of 99 boys (57.6%) and 73 girls (42.4%), with a median age of 6.0 months [IQR: 5.0 to 8.9]. Surgical procedures involved the right lung in 82 cases and the left lung in 90 cases. Complete demographic and clinical characteristics stratified by measurement agreement status are summarized in Table 1.

**Table 1:** Characteristics and respiratory parameters of the 172 infants undergoing one-lung ventilation

Characteristics	Pairs in agreement (*n* = 124)	Pairs not in agreement (*n* = 48)	Statistical test	*P*–value
Sex (boy/girl)	71/53	28/20	χ² test	0.898
Age (month)	6.8 [5.1, 9.7]	5.0 [4.0, 6.4]	Mann–Whitney U	< 0.001
Weight (kg)	8.5 [7.4, 9.8]	7.8 [7.0, 8.5]	Mann–Whitney U	0.014
Hb (g/L)	113.4 ± 9.0	115.3 ± 9.2	Student’s t-test	0.225
Peak airway pressure (cmH₂O)	22 [21, 25]	24 [22, 25]	Mann–Whitney U	< 0.001
Lung compliance (ml/cmH₂O)	3.0 [2.0, 4.0]	3.0 [3.0, 3.0]	Mann–Whitney U	0.003
Tidal volume (ml)	60 [50, 65]	60 [60, 70]	Mann–Whitney U	< 0.001
Tidal volume per kg (ml/kg)	6.8 [5.9, 7.6]	8.2 [7.1, 9.8]	Mann–Whitney U	< 0.001
PEEP level, n (%)			χ² test	< 0.001
0 cmH₂O	13 (10.5%)	48 (100.0%)		
5 cmH₂O	59 (47.6%)	0 (0%)		
7 cmH₂O	52 (41.9%)	0 (0%)		

Values are presented as mean ± standard deviation (SD), median [interquartile range], or number (percentage)

*Abbreviations*: *Hb*Hemoglobin, *PEEP* Positive end–expiratory pressure

### Agreement analysis: TLV

During the TLV phase (T1), Bland-Altman analysis demonstrated a high level of agreement between arterial and end–tidal measurements. The mean bias was 0 mmHg, with 95% limits of agreement (LoA) ranging from − 4.34 to 4.39 mmHg. The 95% confidence intervals (CI) for these limits (-4.91 to 4.96) fell entirely within the pre–defined clinically acceptable range of ± 5 mmHg. Regression analysis confirmed no significant proportional bias (*P* > 0.05 for slope deviation), and the correlation coefficient was robust (*r* = 0.89, *P* < 0.0001) Figure S1.

### Agreement analysis: OLV

**Fig. 2:**
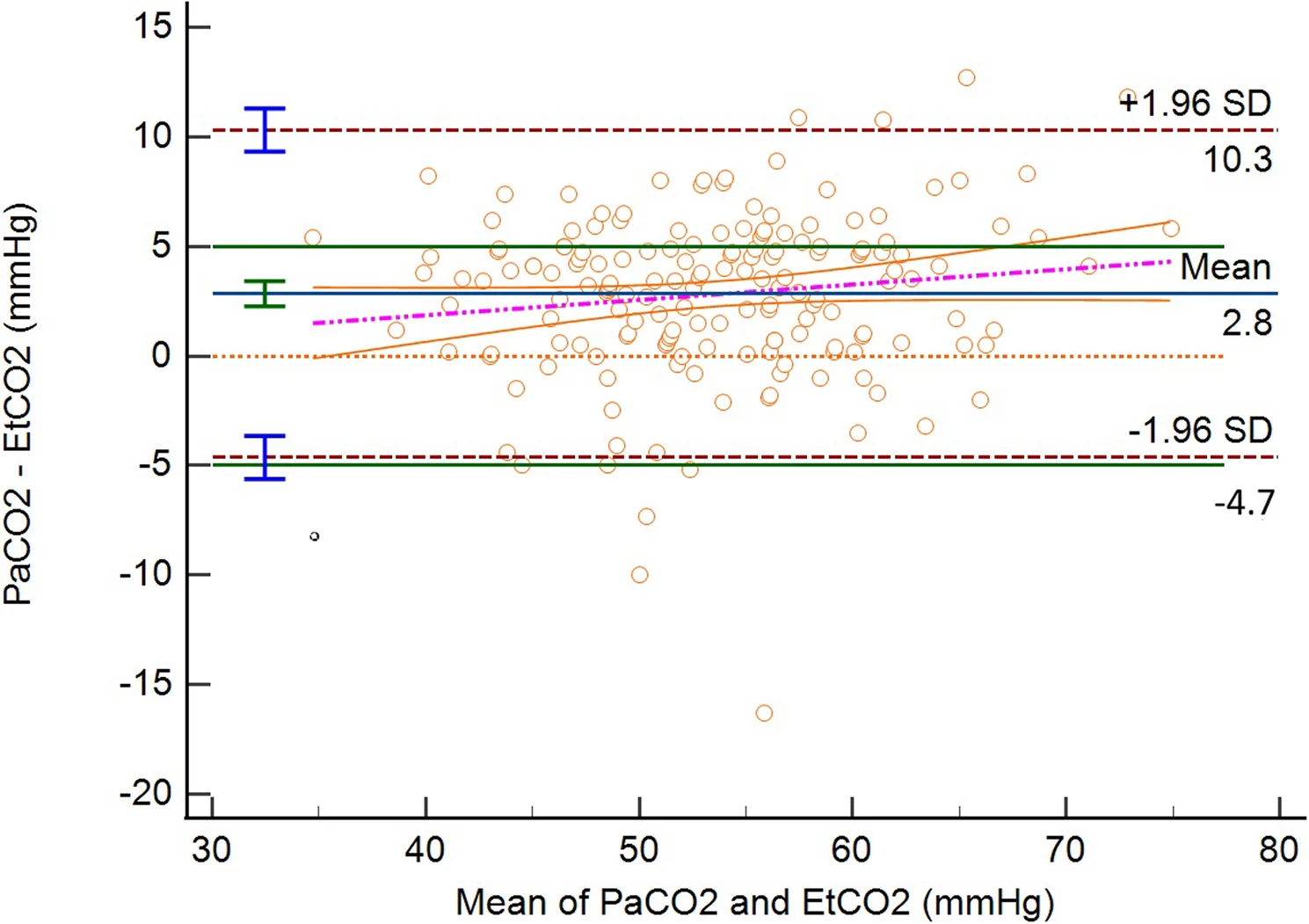
Bland-Altman plot assessing the agreement between arterial (PaCO_2_) and end-tidal (EtCO_2_) carbon dioxide measurements during one-lung ventilation (OLV). Bland–Altman plot comparing PaCO₂ and EtCO₂ during one-lung ventilation. The solid horizontal central line represents the mean bias of 2.8 mmHg. The upper and lower solid blue lines indicate the 95% limits of agreement (LoA), ranging from − 4.7 to 10.3 mmHg. The blue horizontal lines with caps on the left represent the 95% confidence intervals for the bias and the LoA. The pink dashed line represents the linear regression line of the differences against the means, while the solid orange diagonal lines represent the 95% confidence intervals for this regression line. The unit of measurement for both axes is mmHg. The wide distribution of data points outside the clinically acceptable range (± 5 mmHg) illustrates the limited agreement during the OLV phase. The unit of measurement for both the X–axis (mean values) and Y–axis (differences) is millimeters of mercury (mmHg)

In contrast, agreement deteriorated significantly following the transition to OLV (Fig. 2). The mean bias was 2.8 mmHg. The LoA widened to a range of -4.7 to 10.3 mmHg, with the 95% CI extending from − 5.6 to 11.3 mmHg. This interval exceeded the maximum acceptable difference of ± 5 mmHg, indicating that EtCO_2_ cannot reliably replace PaCO_2_ monitoring during OLV.

### Impact of oxygenation on agreement

**Fig. 3:**
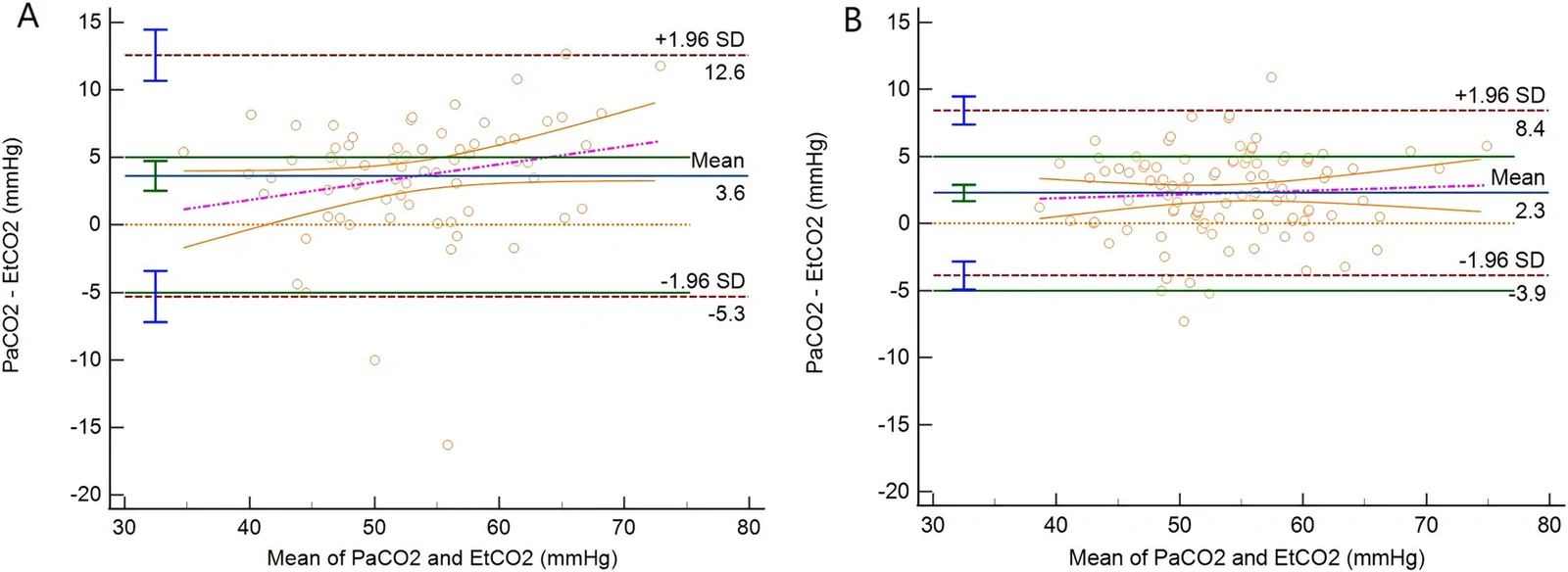
Bland–Altman plots of PaCO2 and EtCO2 agreement during one–lung ventilation, stratified by oxygenation status. **A** Patients with a PaO2/FiO2 ratio <200 mmHg. The mean bias is 3.6 mmHg, with wide 95% limits of agreement (LoA) ranging from -5.3 to 12.6 mmHg, indicating poor agreement in patients with compromised oxygenation. **B** Patients with a PaO2/FiO2 ratio ≥200 mmHg. The mean bias is 2.3 mmHg, with narrower 95% LoA ranging from -3.9 to 8.4 mmHg. In both plots, the central solid dark blue line represents the mean bias, surrounded by its 95% confidence interval (CI) indicated by the green dashed lines and shaded area. The dashed maroon lines represent the 95% LoA, while the blue shaded regions bounded by solid blue lines indicate the respective 95% CIs for these limits. The pink dashed diagonal line represents the regression line of the differences against the means, and the solid orange diagonal lines represent the 95% CI for this regression line. The unit of measurement for both the X–axis (mean values) and Y–axis (differences) is millimeters of mercury (mmHg). (Note: Please be aware of the different Y–axis scales between subplot A and subplot B, which were retained to preserve the optimal visual distribution of the data points)

Oxygenation status significantly influenced the agreement between PaCO₂ and EtCO₂. Bland-Altman analysis showed that patients with a PaO₂/FiO₂ ratio < 200 mmHg (Fig. 3A) had a larger mean bias (3.6 mmHg) and wider limits of agreement (− 5.3 to 12.6 mmHg) compared with those having a ratio ≥ 200 mmHg (mean bias 2.3 mmHg; limits of agreement − 3.9 to 8.4 mmHg; Fig. 3B). Furthermore, the proportion of measurements within the clinically acceptable range of ± 5 mmHg was significantly lower in the low–oxygenation group (55.9% vs. 81.7%, *P* = 0.0001). It should be noted that this ± 5 mmHg range reflects a clinical decision threshold rather than physiological equivalence, as positive and negative gradients likely represent distinct underlying mechanisms.

### Method comparison regressions

Despite the wide limits of agreement observed during OLV, regression analyses confirmed a strong linear relationship between PaCO₂ and EtCO₂. Deming regression analysis demonstrated a strong linear relationship between PaCO₂ and EtCO₂, yielding the equation y = − 4.970 + 1.149x (Figure S2-A). The 95% confidence interval (CI) for the intercept (− 10.36 to 0.42) included 0, indicating no significant constant bias, whereas the 95% CI for the slope (1.05 to 1.25) did not include 1, suggesting the presence of proportional bias. Furthermore, the residual plot (Figure S2–B) showed a random distribution of residuals around the zero line, supporting the appropriateness of the linear model.

### Estimated dead-space index and correlation

The estimated dead–space index, calculated as (PaCO₂–EtCO₂)/PaCO₂, increased significantly from a median of 3.88% [IQR: 2.28–5.47] during TLV to 6.89% [IQR: 2.94–9.83] during OLV (*P* < 0.0001). This index should be interpreted as a clinical surrogate reflecting changes in ventilation efficiency rather than as a true measurement of physiological dead space, since mixed expired CO₂ (PECO₂) was not available in our setup.

Spearman’s rank correlation analysis between continuous P/F ratio and the EtCO₂–PaCO₂ gradient showed a significant negative correlation (ρ = -0.219, *P* = 0.0039).

The Spearman analysis demonstrated a much stronger and highly significant positive correlation between the continuous OI and the EtCO₂–PaCO₂ gradient (Spearman’s ρ = 0.211, *P* = 0.005). This continuous modelling robustly confirms our initial finding: as the oxygenation index worsens, the CO₂ gradient significantly widens.

### Predictors of disagreement

In the multivariable logistic regression analysis, three protocol–related variables were excluded due to structural confounding inherent to the secondary analysis design. PEEP and VT/kg were excluded because all 48 mismatched pairs (100%) occurred under ZEEP with VT ≥ 6 mL/kg while no mismatches occurred under applied PEEP with VT ≤ 6 mL/kg, resulting in quasi-complete separation and perfect collinearity with randomized arm allocation. Peak airway pressure was excluded due to multicollinearity with lung compliance. The final model included patient–level covariates that varied within randomized strata: lung compliance, age, sex, surgical side, and intraoperative hypotension (Table 2). Higher lung compliance was independently associated with greater odds of EtCO₂–PaCO₂ agreement (adjusted OR 1.66 per mL/cmH₂O increase, 95% CI 1.05–2.64, *P* = 0.030). No other covariate reached statistical significance.

**Table 2:** Multivariate logistic regression analysis for factors associated with EtCO₂–PaCO₂ Agreement

Variables	Adjusted OR	95% CI	*P*–value
Lung compliance (ml/cmH₂O)	1.66	1.05, 2.64	0.03
Age (months)	1.1	0.98, 1.24	0.102
Sex (Boy vs. Girl)	1.11	0.55, 2.24	0.772
Surgical side (Right vs. Left)	1.37	0.68, 2.74	0.379
Hypotension (Yes vs. No)	1.03	0.47, 2.24	0.94

PEEP, peak airway pressure, and weight-adjusted tidal volume (VT/kg) were excluded from the multivariable model. PEEP and VT/kg were excluded due to quasi-complete separation with the outcome (all 48 mismatched pairs occurred under ZEEP with VT = 8 mL/kg, while no mismatches occurred under applied PEEP with VT ≤ 6 mL/kg) and perfect collinearity with randomized arm allocation in the parent trial (ZEEP was protocol-paired with 8 mL/kg VT, while applied PEEP was protocol–paired with 6 mL/kg VT). Peak airway pressure was excluded due to mathematical multicollinearity with lung compliance

Additionally, continuous analysis of the PaCO₂−EtCO₂ gradient demonstrated a right-shifted and broader distribution in the non-protective ventilation group compared with the protective ventilation group (Figure S3; Mann–Whitney U test, *P* < 0.001). Notably, all gradients exceeding the predefined clinical threshold of > 5 mmHg occurred in the non-protective ventilation group.

## Discussion

### Principal findings

In this secondary analysis of a randomized trial of lung-protective ventilation in infants undergoing one-lung ventilation (OLV), EtCO₂ showed a linear relationship with PaCO₂ but demonstrated insufficient agreement to serve as a stand-alone substitute for arterial blood gas analysis. This finding is consistent with previous pediatric and neonatal studies showing that EtCO₂ may correlate with PaCO₂ but may not reliably predict arterial CO₂ under conditions of altered ventilation–perfusion matching, small tidal volumes, or infant-specific respiratory mechanics [5–8, 13]. Clinically relevant EtCO₂–PaCO₂ disagreement, defined as PaCO₂−EtCO₂ >5 mmHg, occurred in 48 of 172 paired measurements and was observed exclusively in infants ventilated with the non-protective strategy.

### Two main findings deserve emphasis

First, the lung-protective ventilation strategy, as a composite intervention combining lower tidal volume and applied PEEP, was associated with markedly improved EtCO₂–PaCO₂ agreement. No disagreement events occurred in the protective ventilation arm. This observation is consistent with prior pediatric and adult OLV studies suggesting that lung-protective ventilation and appropriate PEEP can improve oxygenation, respiratory mechanics, and ventilation–perfusion matching during thoracic surgery [12, 20–22]. However, because tidal volume and PEEP were jointly assigned in the parent trial, their independent contributions cannot be separated in the present analysis.

Second, lower lung compliance was independently associated with widened EtCO₂–PaCO₂ gradients, suggesting that impaired respiratory mechanics may identify infants in whom EtCO₂ is more likely to underestimate PaCO₂. This is physiologically plausible because reduced compliance may reflect decreased aerated lung volume, atelectasis, or increased ventilation–perfusion heterogeneity, all of which may widen the arterial-to-end-tidal CO₂ gradient [15, 17, 23].

These findings have practical implications. During infant OLV, EtCO₂ should be interpreted cautiously, particularly when lung compliance is reduced, oxygenation is impaired, or non-protective ventilatory settings are used. In such situations, intermittent arterial blood gas analysis remains necessary to confirm adequacy of ventilation, especially given the potential clinical consequences of unrecognized hypercapnia in neonates and infants [1–4].

### Physiological interpretation

A positive PaCO₂–EtCO₂ gradient is physiologically expected because EtCO₂ reflects alveolar gas sampled at the airway opening, whereas PaCO₂ reflects arterial CO₂ tension influenced by global ventilation–perfusion matching, dead space, and shunt [15–17, 24, 25]. This baseline gradient may be amplified during infant OLV. Infants have a highly compliant chest wall, low functional residual capacity, and a tendency toward airway closure and atelectasis during anesthesia [10, 17, 20]. In the lateral decubitus position, collapse of the operative lung and compression of the dependent lung may further worsen ventilation–perfusion mismatch [10, 17].

Our finding that reduced lung compliance was associated with widened PaCO₂–EtCO₂ gradients is consistent with this physiology. Poor compliance may indicate atelectasis, airway closure, or reduced effective alveolar ventilation, which can increase heterogeneity of ventilation and perfusion and reduce the reliability of EtCO₂ as a surrogate for PaCO₂ [15, 17, 20]. However, these mechanisms were not directly measured in this study. Therefore, they should be interpreted as plausible explanations rather than proven causal pathways.

The exclusive occurrence of disagreement in the non-protective ventilation arm also supports the possibility that higher tidal volume without PEEP may worsen ventilation–perfusion mismatch in the infant lung. PEEP may help preserve dependent-lung recruitment, while lower tidal volume may reduce regional overdistension and ventilation heterogeneity [12, 20–22]. Nevertheless, because tidal volume and PEEP were assigned together, the present study cannot determine whether improved agreement was due to PEEP application, tidal volume reduction, or their combined effect as a composite lung-protective strategy.

### Negative gradients

A subset of paired measurements showed negative gradients, in which EtCO₂ exceeded PaCO₂. Mild negative gradients have been previously recognized and may occur under conditions of rapid physiological change, altered CO₂ transport, changes in pulmonary blood flow, or technical limitations of capnography and blood gas sampling [24, 25]. In infant thoracoscopic surgery, CO₂ insufflation may further complicate interpretation because absorbed CO₂ can alter systemic CO₂ load and end-tidal measurements [26].

However, large negative gradients are less likely to be purely physiological. Potential technical contributors include timing differences between arterial sampling and capnography recording, pre-analytical blood gas errors, difficulty obtaining a stable alveolar plateau in infants with small tidal volumes, and airway leak or sampling-line artifacts [6–8, 24, 25]. Therefore, negative gradients, particularly extreme values, should be interpreted cautiously. This reinforces the broader conclusion that EtCO₂ alone may be insufficient for precise assessment of PaCO₂ during infant OLV.

### Comparison with previous studies

Our findings are consistent with previous pediatric and neonatal studies showing limited agreement between end-tidal and blood CO₂ measurements. Badgwell et al. demonstrated that end-tidal CO₂ measurements in infants and children may vary depending on sampling site and airway factors [6]. Kugelman et al. similarly showed that distal capnography may improve estimation of arterial CO₂ in intubated infants but does not eliminate disagreement [7]. Tingay et al. reported that end-tidal CO₂ may be clinically useful in ventilated postsurgical neonates, although reliability depends on patient and ventilatory conditions [13]. In contrast, Karlsson et al. reported poor performance of mainstream capnography in newborn infants during general anesthesia [27].

The variation among studies likely reflects differences in patient age, lung mechanics, ventilation mode, surgical setting, capnography technique, and definitions of acceptable agreement. By focusing specifically on infants undergoing OLV, the present study extends prior work to a high-risk physiological setting in which shunt, atelectasis, and ventilation–perfusion mismatch are particularly prominent [10, 17].

The use of a ± 5 mmHg threshold was chosen for clinical interpretability and comparability with previous capnography literature. However, this symmetric threshold is a simplification. Physiologically, PaCO₂ is generally expected to exceed EtCO₂ because of dead space and ventilation–perfusion mismatch [15–17, 24, 25]. Accordingly, our additional continuous-gradient analyses were included to avoid overreliance on dichotomized agreement alone. Methodologically, agreement analysis in studies with repeated paired measurements should also account for within-subject clustering, as emphasized by Bland and Altman [18].

### Protective ventilation and clinical implications

A clinically important observation of this study is that lung-protective ventilation was associated with improved PaCO₂–EtCO₂ agreement. Although the mechanism cannot be directly established from our data, the combination of lower tidal volume and PEEP may reduce atelectasis, improve dependent-lung ventilation, and attenuate ventilation–perfusion mismatch during OLV [12, 20–22]. Previous studies in pediatric thoracic surgery have shown that lung-protective ventilation can improve perioperative respiratory outcomes [12, 20], while physiological studies during OLV have demonstrated that PEEP can improve oxygenation and respiratory mechanics by modifying ventilation–perfusion relationships [21, 22].

For clinicians, these results suggest that EtCO₂ values should not be interpreted in isolation during infant OLV. A widened PaCO₂–EtCO₂ gradient is more likely when lung compliance is poor, oxygenation is impaired, or non-protective ventilatory settings are used. Under these circumstances, arterial blood gas confirmation is particularly important. Conversely, lung-protective ventilation may improve not only oxygenation and respiratory mechanics but also the reliability of EtCO₂ as a continuous non-invasive monitoring tool.

### Strengths and limitations

This study has several strengths, including a homogeneous infant OLV population, protocolized ventilation derived from a randomized trial, and temporally paired PaCO₂ and EtCO₂ measurements. These features strengthen the internal validity of the agreement analysis.

Several limitations should also be acknowledged.

First, this was a single-center secondary analysis involving infants younger than two years; therefore, generalizability to older children or other clinical settings may be limited.

Second, tidal volume and PEEP were assigned together in the parent trial, and all disagreement events occurred in the non-protective arm. This structural collinearity prevented separate estimation of the independent effects of tidal volume, PEEP, and airway pressure. Future factorial-design studies would be required to determine the relative contribution of each ventilatory component.

Third, although lung compliance was associated with disagreement, the study did not directly measure atelectasis, regional ventilation, pulmonary blood flow, or true physiological dead space. The dead-space index used in this study was a clinical surrogate based on PaCO₂ and EtCO₂ rather than a direct volumetric capnography measurement. Because true dead-space assessment requires measurement of mixed expired CO₂, mechanistic interpretations regarding alveolar dead space and ventilation–perfusion mismatch remain inferential [15, 17].

Fourth, the dichotomous > 5 mmHg threshold, while clinically practical, inevitably loses information compared with continuous modeling and does not fully reflect the physiological asymmetry of PaCO₂–EtCO₂ gradients. Future studies should consider both continuous gradient analysis and directional thresholds. Finally, technical factors such as sampling delay, capnograph response time, airway leak, small tidal volumes, and blood gas handling may have contributed to some extreme gradient values, particularly negative gradients [6–8, 24, 25].

## Conclusion

EtCO₂ monitoring during infant OLV correlates with PaCO₂ but does not provide sufficiently reliable agreement to replace arterial blood gas analysis. Lung-protective ventilation, as a combined strategy of lower tidal volume and PEEP, was associated with substantially improved PaCO₂–EtCO₂ agreement, whereas reduced lung compliance identified infants at higher risk of clinically relevant disagreement. During infant OLV, especially in the presence of poor compliance or impaired oxygenation, EtCO₂ should be interpreted cautiously and supplemented by arterial blood gas measurement when accurate assessment of ventilation is required.

## Supplementary Information

Supplementary Material 1.

## Data Availability

The datasets generated and/or analyzed during the current study are not publicly available due to patient privacy but are available from the corresponding author on reasonable request.
