# Supplementary material for: Reliability of end-tidal carbon dioxide as a surrogate for arterial carbon dioxide assessment in infants undergoing thoracoscopic surgery with one-lung ventilation
Source: BMC Anesthesiol. 2026 Jul 3;26:576. doi: 10.1186/s12871-026-04077-3 (PMC13602595; doi:10.1186/s12871-026-04077-3)
Supplement: Supplementary file 1 [file 12871_2026_4077_MOESM1_ESM.docx]

**Figure S1. Bland–Altman analysis of PaCO₂–EtCO₂ agreement during two-lung ventilation.** The y-axis represents the PaCO₂–EtCO₂ difference, and the x-axis represents the mean of PaCO₂ and EtCO₂. The central solid line indicates the mean bias, and the upper and lower solid lines indicate the 95% limits of agreement. During two-lung ventilation, PaCO₂ and EtCO₂ showed close agreement, with a mean bias of approximately 0.0 mmHg and 95% limits of agreement from −4.3 to +4.4 mmHg. The same axis scale was used as in the corresponding OLV Bland–Altman plot to allow direct visual comparison.

**Figure S2. Deming regression analysis comparing PaCO₂ and EtCO₂ during one-lung ventilation.**

*(A) Residual plot showing the distribution of differences between the observed PaCO₂ values and the Deming regression line. The residuals are randomly scattered around the solid blue horizontal zero line, indicating an adequate model fit.*

*(B) Scatter diagram with the Deming regression line. The solid dark blue line represents the regression equation (y = −4.970 + 1.149x; n = 172). The dashed maroon line represents the line of identity (y = x).*

**Figure S3. Ventilation Strategy–Stratified Distribution of PaCO₂–EtCO₂ Gradients.**

*Continuous analysis of the PaCO₂−EtCO₂ gradient demonstrated a right-shifted and broader distribution in the non-protective ventilation group compared with the protective ventilation group (Figure S3; Mann–Whitney U test, p<0.001). Notably, all gradients exceeding the predefined clinical threshold of >5 mmHg occurred in the non-protective ventilation group.*
